# Prediction of Myopia Among Undergraduate Students Using Ensemble Machine Learning Techniques

**DOI:** 10.1002/hsr2.70874

**Published:** 2025-05-26

**Authors:** Isteaq Kabir Sifat, Tajin Ahmed Jisa, Jyoti Shree Roy, Nourin Sultana, Farhana Hasan, Md Parvez Mosharaf, Md. Kaderi Kibria

**Affiliations:** ^1^ Department of Statistics Hajee Mohammad Danesh Science and Technology University Dinajpur Rangpur Bangladesh; ^2^ Department of Statistics University of Rajshahi Rajshahi Bangladesh; ^3^ School of Business, Faculty of Business, Education, Law and Arts University of Southern Queensland Toowoomba Queensland Australia

**Keywords:** Dinajpur city, ensemble machine learning, Myopia, refractive error, SHAP analysis, undergraduate students

## Abstract

**Background and Aims:**

Myopia is a prevalent refractive error, particularly among young adults, and is becoming a growing global concern. This study aims to predict myopia among undergraduate students using ensemble machine learning techniques and to identify key risk factors associated with its development.

**Methods:**

A cross‐sectional study was conducted in Dinajpur city, collecting 514 samples through a self‐structured questionnaire covering demographic information, myopia prevalence and risk factors, knowledge and attitudes, and daily activities. Four feature selection techniques Boruta‐based feature selection (BFS), Least Absolute Shrinkage and Selection Operator regression, Forward and Backward Selection and Random Forest (RF) identified 12 key predictive features. Using these features, ensemble methods, including logistic regression artificial neural network, RF, Support Vector Machine, extreme gradient boosting, and light gradient boosting machine were employed for prediction. Model performance was evaluated using accuracy, precision, recall, F1‐score, and area under the curve (AUC).

**Results:**

The stacking ensemble model achieved the highest performance, with an accuracy of 95.42%, recall of 93.42%, precision of 98.85%, F1‐score of 96.08%, and AUC of 0.979. SHapley Additive exPlanations analysis identified key risk factors, including visual impairment, family history of myopia, excessive screen time, and insufficient outdoor activities.

**Conclusion:**

These findings demonstrate the effectiveness of ensemble machine learning in predicting myopia and highlight the potential for early intervention strategies. By identifying high‐risk individuals, targeted awareness programs and lifestyle modifications can help mitigate myopia progression among undergraduate students.

**Abbreviations**
ANN: artificial neural network
AUC: area under the curve
BFS: boruta‐based feature selection
DGC: Dinajpur Government College
EFB: exclusive feature bundling
FBS: forward and backward selection
HSTU: Hajee Mohammad Danesh Science and Technology University
LASSO: least absolute shrinkage and selection operator
LGB: light gradient boosting machine
LR: logistic regression
MARMC: M Abdur Rahim Medical College
MICE: multiple imputation by chained equations
RF: random forest
SHAP: shapley additive explanations
SVM: support vector machine
XGB: extreme gradient boosting

## 1 Introduction

Myopia, or nearsightedness, is a common refractive error of the eye that has become a growing global health concern, particularly among young adults [1, 2]. Its prevalence is alarmingly high in academic populations, including undergraduate students, who are frequently exposed to prolonged near‐work activities and digital screen usage [3, 4]. The “myopia boom” in the twenty‐first century has raised serious global concerns, especially in East Asia, where 80–90% of 18‐year‐olds are myopic [5, 6]. Myopia is associated with an increased risk of serious complications such as retinal detachment, glaucoma, and cataracts, making it a pressing public health issue [7]. The highest prevalence of myopia is reported among Asians aged 20 to 29 years, with rates reaching 47.3% [8]. In Bangladesh, a study found a myopia prevalence of 22.1% [9]. By 2050, it is projected that nearly 50% of the world's population, approximately 5 billion people, will be affected by myopia [10]. If left unmanaged, myopia can lead to severe complications, including visual impairment, reduced quality of life, and an elevated risk of ocular diseases [4, 11, 12]. Early identification and intervention are critical to mitigating its progression.

Several factors contribute to the development of myopia, including genetic predisposition, environmental influences, and lifestyle habits [13, 14, 15]. Undergraduate students are particularly vulnerable due to their academic routines, which often involve extended near‐work activities, prolonged screen exposure, and limited outdoor engagement are the key risk factors for myopia [16]. While these factors are well recognized, predicting myopia using a practical, scalable, and accessible approach remains a challenge. Traditional clinical methods, such as fundus imaging and refractive error measurement, are reliable but require specialized equipment and trained professionals, limiting their widespread applicability. To address this gap, a questionnaire‐based screening method combined with ML offers a cost‐effective alternative for early detection, particularly in resource‐limited settings.

Machine learning (ML) has emerged as a powerful tool in developing predictive models for myopia and high myopia across different populations [17, 18, 19, 20]. Previous studies have demonstrated the effectiveness of various ML models, including RF, K‐Nearest Neighbors (KNN), SVM, LR, BP Neural Network, and Naive Bayes (NB), with SVM achieving accuracies as high as 93% [17, 21, 22, 23, 24]. Another study utilized Kernel SVM, Decision Tree, RF, KNN, and NB to detect pathologic myopia, with Kernel SVM showing an accuracy of 91.47% [25]. While ML has been extensively applied to image‐based myopia detection, its use in predicting myopia based on behavioral and lifestyle factors remains underexplored. This study bridges that gap by utilizing a questionnaire‐based approach combined with ensemble ML models to predict myopia risk in undergraduate students. To enhance predictive performance, four feature selection methods BFS, LASSO regression, FBS, and RF were employed to identify the most relevant features, leveraging their complementary strengths. By integrating these diverse techniques, the study ensures a balanced approach that captures both linear and nonlinear relationships within the data set.

This study focuses on predicting myopia among undergraduate students using ensemble ML techniques and identifying key risk factors associated with its development. By leveraging self‐reported lifestyle and behavioral data, the research provides a framework for early myopia detection that is accessible, scalable, and applicable in large populations where clinical diagnostic tools may not be readily available. The findings are expected to support data‐driven interventions, promote awareness, and guide public health strategies for mitigating myopia prevalence in academic populations.

## 2 Materials and Methods

### 2.1 Study Design, Sample Size and Sampling Technique

A cross‐sectional study was conducted among undergraduate students in Dinajpur city, located in northern Bangladesh, from May 17, 2024 to June 17 2024. The study focused on students from three institutions: Hajee Mohammad Danesh Science and Technology University (HSTU), Dinajpur Government College (DGC), and M Abdur Rahim Medical College (MARMC). These institutions were selected to ensure diversity among students from different academic backgrounds, including science and technology, medical sciences, and general education. This approach aimed to provide a broad perspective on myopia prevalence among undergraduate students in Dinajpur. Only undergraduate students who willingly participated in the survey were considered. Those who were not currently full‐time students or declined to participate were excluded. The required sample size was determined using Cochran's single proportion formula [26] ($n=Z^{2}p\left(1-p\right)/e^{2}$), where *n* is the required sample size (which is 514), *Z*‐score is 1.96, estimated proportion (*p*) is 0.5 and margin of error (*e*) is 2.205%. We successfully collected 514 samples from all participants.

To achieve a sample size of 514, we used stratified sampling, dividing the participants into separate strata from three institutes: HSTU, DGC, and MARMC. From each stratum, participants were randomly selected using simple random sampling (SRS). This approach minimizes selection bias and ensures that each potential participant within a stratum has an equal chance of being chosen. The number of participants selected from each institute (stratum) was proportionate to its representation in the total population of undergraduate students from these institutes. Since the complete number of undergraduate students across these institutions was not available, we could not determine an exact response rate. Participation was voluntary, and no incentives were provided.

### 2.2 Questionnaire Design, Data Collection and Processing

We developed a self‐structured questionnaire based on a comprehensive literature review [9, 17, 18, 19, 20, 21] and consultation with experts in the field of myopia and vision‐related research. Although the questionnaire was not adapted from a previously validated instrument, a pilot study was conducted before the main data collection. The pilot study aimed to assess the clarity, relevance, and comprehensiveness of the questions and allowed for refinement of the questionnaire to improve its structure and relevance for the target population. Feedback from the pilot study was used to adjust ambiguous or unclear questions, ensuring better data quality. The questionnaire was designed to gather information on (i) demographic details, (ii) prevalence and risk factors of myopia, (iii) knowledge and attitudes toward myopia, and (iv) daily activities potentially influencing myopia development. Printed copies of the questionnaire were distributed to respondents, who were given enough time to complete it. Before distribution, participants were briefed on the study's objectives, and written consent was obtained from each participant. During data processing, we identified and addressed missing values using Multiple Imputation by Chained Equations (MICE). Additionally, we assessed multicollinearity among predictor variables using variance inflation factors (VIF) where all VIF value being less than 5.

#### 2.2.1 Outcome Variable

The outcome variable for this study is the myopia status of the students. It was determined through self‐reported information, supplemented by medical records when available. This variable is classified into two categories: (i) “Yes” (coded as 1) and (ii) “No” (coded as 0).

#### 2.2.2 Independent Variables

Demographic variables included age, gender, residence (urban/rural), living conditions (hall, mess, home, other), and economic status (poor, middle‐income, rich). Health‐related variables included family history of myopia, premature birth, eye pain, headaches, visual stress, eye redness, severe eye injury, and conditions like glaucoma and retinal detachment. Family history of myopia, eye injury, eye disorder, glaucoma, and retinal detachment were categorized as “yes” or “no” [9]. Responses to eye pain were categorized as “yes,” “no,” or “maybe.” Symptoms of headaches and visual stress (e.g., blurred vision, eye strain) were categorized as “yes,” “no,” or “sometimes.” Visual impairment was categorized as “no,” “mild,” “moderate,” “severe,” or “blind.” Screen light exposure and near‐work activities were categorized as “yes,” “no,” or “maybe,” based on participant awareness and reported habits. A study have used similar categories to assess self‐reported sreen light exposure [9]. Time spent on outdoor activities and electronic devices, as well as involvement in sports and mobile phone usage, were categorized according to specific thresholds: for outdoor activities (< 0.5 h, 0.5–1 h, 1–2 h, > 2 h), electronic device use (> 6 h/day or less), and mobile phone use (greater than 4 h or less). Dietary intake was categorized as “poor,” “middle,” or “rich”. All of the categories of the variables were determined based on the literature [9, 17, 18, 19, 20, 21].

#### 2.2.3 Feature Selection

Feature selection approaches play a crucial role in machine learning processes because they make it possible to extract the most pertinent features for classification [22, 23]. In our study, we employed four feature selection algorithms such as BFS method, LASSO regression, FBS, and RF to identify the most significant subset of features. BFS, a wrapper‐based feature selection method, was selected due to its robustness and ability to identify all relevant features, even those that might be overlooked by other methods. It operates by iteratively comparing feature importance scores using the random forest classifier algorithm, ensuring that only statistically significant features are retained [24]. We included FBS as a stepwise method because it provides a systematic approach to refining the feature set by iteratively adding or removing one variable at a time, improving the model's generalization ability [25]. The RF algorithm was chosen due to its power in evaluating feature importance by constructing numerous decision trees. This method ranks features based on their contribution to reducing impurity, making it a reliable technique for identifying features that most influence the outcome [27]. Lastly, LASSO regression was included to address overfitting by applying L1 regularization, which penalizes the inclusion of irrelevant or redundant features in the model. This feature selection method is effective when dealing with high‐dimensional data, as it encourages sparsity and enhances model interpretability [28]. To determine the most important risk factors connected to myopia, we combined the outcomes of all four feature selection approaches after applying each one.

$$\mathrm{Important features}=\overset{r}{\underset{i=1}{⋃}}{\mathrm{Identified features form myopia dataset}}_{i}$$

Where, in this case, *r* = 4, denotes the number of feature selection techniques used. This comprehensive approach ensures that we consider various perspectives and methodologies in identifying the key features, thereby enhancing the reliability and robustness of our findings.

## 3 Machine Learning Algorithms

### 3.1 Artificial Neural Network

ANN is a mathematical model based on the functional characteristics of biological neural networks [29]. It is made up of interconnected processing nodes arranged into three categories: hidden, output, and input layers. The hidden layer is connected to the output, while the input layer is connected to the hidden layer with the updated weight [30]. The BP training algorithm is a ramp descent algorithm. The BP method is used to improve network performance by minimizing total error by adjusting the weights on the ramp. Training is discontinued when the mean square error (MSE) values stop declining and begin to rise, indicating over‐training [31, 32].

$$\mathrm{MSE}\%=1/n\sum_{i=1}^{n}{\left(d_{i}-O_{i}\right)}^{2}$$

In this case, the output data number is n, the network output value is O_i_, the target or true value is d_i_, and each other represents the network output value.

### 3.2 Random Forest

RF is an ensemble machine learning method that uses decision trees as its base classifier. Breiman [33] proposed RF, an ensemble of tree‐based predictors in which each tree is trained with values from a random vector sampled independently and with the same distribution as the other trees in the forest [34]. In theory, the *k*th tree is trained with a random vector $\Theta_{k}$, which has the same distribution as the previous random vectors $\Theta_{1}$ to $\Theta_{k-1}$, but is independent of them. This yields a tree ℎ(*X*, $\Theta_{k}$), where *X* is an input vector. The average predictions of many trees planted in the forest are obtained, increasing prediction accuracy and preventing over‐fitting. Mathematically,

$$\hat{Y}=1/n\sum_{k=1}^{n}h_{k}\left(X\right)$$

when 1 ≤ *k* < *n*, *n* is the total number of trees formed, and $\hat{Y}$ is the target.

$$E_{X,Y}{\left(Y-h\left(X\right)\right)}^{2}$$

for the input vector *X* and the target *Y* gives the mean‐squared generalization error of any tree ℎ(*X*). Since there are an infinite number of trees in the forest, the

$$E_{X,Y}{\left(Y-{\mathrm{av}}_{k}h\left(X,\Theta_{k}\right)\right)}^{2}\to E_{X},Y{\left(Y-E_{\theta }h\left(X,\Theta \right)\right)}^{2}$$

### 3.3 Logistic Regression

LR is another type of supervised learning approach. It is a statistical model. With logistic regression, the target value's likelihood is predicted. There are two categories for the target characteristic: success and nonsuccess. When it succeeds, it yields 1, and when it fails, it yields 0.

$$P=\frac{1}{1+\text{exp}\left(b_{0}+b_{1}x+b_{2}x^{2}\right)}$$

Where *x* is a variable that represents a logistic regression, b_0_, b_1_, and b_2_ are biases, and *P* is the predicted value. Applications for machine learning in social science and medicine include spam recognition, diabetes diagnosis, cancer detection, and more [35].

### 3.4 Support Vector Machine

Corinna, Cortes, and Vapnik introduced the concept of support vector machine (SVM) for the first time in 1995 [36]. A supervised machine learning method called Support Vector Machine (SVM) is frequently applied to pattern recognition and classification issues. The SVM algorithm carries out a classification by building a multidimensional hyperplane that maximizes the margin between two data clusters to provide the best possible discrimination between two classes [37]. SVM maximizes the geometric margin while also minimizing the empirical classification error. Therefore, SVM stands for Maximum Margin Classifiers. The kernel trick, which enables classifier construction without explicit knowledge of the feature space, is a technique used by SVMs to efficiently perform nonlinear classification [38]. The mathematical theory of SVMs is briefly summarized here [39]. Consider a binary classification task with a set of training samples that are linearly separable.

$$S=\left\{\left(x_{1}{,y}_{1}\right)\;\text{…}.\;\left(x_{m}{,y}_{m}\right)\right\}\;,$$

where $y_{i}$ is the class label and $x\in R^{d},$ meaning that x is in a d‐dimensional input space; that is, $y_{i}\in \left\{-1,1\right\}$. The class to which the data belongs is indicated by the label. One may therefore define an appropriate discriminating function as follows:

f(x)=sgn({w.x}+b).

Vector *w* controls the orientation of a discriminant plane (or hyperplane), and the bias or offset is represented by vector b. The inner product of vectors w and x is denoted as {*w.x*}. It is obvious that the training data can be correctly classified by an infinite number of different planes.

### 3.5 Extreme Gradient Boosting

XGB is an effective ensemble‐based machine learning algorithm. A group of base classifiers make up the XGBoost algorithm, a class of lifting algorithms. The idea is to create several sub‐data sets from the original data set. The base classifier randomly assigns each sub‐data set to predict; the base classifier's output is then weighted; the end result is the sum of the predictions made by the weak classifiers [40]. A learning algorithm called “boosting” looks to build a strong classifier from weaker classifiers or learners. Both the strong and weak classification models make reference to the relationship between the expected and actual classes. The subsequent classifier can alter the errors of the preceding one by iteratively stacking classifiers on top of one another. Until the training data set correctly predicts the target variable's membership class label, this process is repeated [41]. The tree‐derived predictions can be expressed mathematically as follows

$$\hat{y}=\phi \left(x\right)=\frac{1}{n}\sum_{k=1}^{n}f_{k}\left(x\right)$$

where *Ŷ* is the predicted *ETo*, 1 ≤ *k* ≤ *n*, and A total of *n* functions is what the *n* number of trees have learned. The set of functions *f*_k_ that are employed in the model is discovered by minimizing the following regularized objective L(ϕ):

$$L\left(\phi \right)=\sum_{i}l\left(\hat{y_{i}},y_{i}\right)+\sum_{k}\Omega \left(f_{K}\right)$$

### 3.6 Light Gradient Boosting Machine

A GB system called LGBM makes use of tree‐based learning methods. Two cutting‐edge methods, Exclusive Feature Bundling (EFB) and Gradient‐based One‐Side Sampling (GOSS), are used in its design to make it efficient and dispersed [42]. LGBM's main advantage is a significant speedup of the training process, which frequently yields a more efficient model. Using *n* estimator's numbers of boosted trees LGBM is built atop decision tree algorithms. For prediction tasks, tree boosting algorithms perform better than others [43, 44].

To enhance predictive accuracy in myopia classification, we employed multiple machine learning models, including logistic regression (LR), artificial neural networks (ANN), random forest (RF), support vector machines (SVM), extreme gradient boosting (XGB), and light gradient boosting machine (LGBM). Each model was selected for its unique strengths LR for linear relationships, ANN and SVM for capturing complex nonlinear patterns, and RF and boosting models for feature importance and handling high‐dimensional data.

### 3.7 Stacking Model

A stacking ensemble approach was adopted to leverage the complementary strengths of these models, improving overall prediction accuracy and generalization [34]. Stacking integrates multiple optimized base models and uses their predictions as input for a meta‐learner, mitigating individual model limitations. This method is particularly beneficial for handling noisy datasets, as it optimally combines diverse models, assigns appropriate weights based on performance, and reduces the impact of noise in predictions [45, 46, 47]. In this study, a stacking classifier was built with LR, ANN, RF, SVM, XGB, and LGBM as base classifiers at level 0, and LR as the meta‐learner at level 1. The stacking model that is being suggested utilizes the prediction outputs from the level 0 classifiers, which are then sent to the level 1 classifier for final prediction.

### 3.8 Cross Validation and Tune Hyperparameters

The ML methods covered above have extra parameters, sometimes known as hyperparameters (see Table 1). The user can explicitly define hyperparameters before the learning process begins to enhance the model's performance. Using a recurring 10‐fold (*K*10) cross‐validation process, the grid search technique's hyperparameter values in the training set were modified. A training subset and a verification set are divided from the training data set in a 7:3 ratio to apply the *K*10 approach.

**Table 1:** The hyperparameter value in machine learning models.

Models	Hyperparameter
RF	mtry = (1, 2, 3, 4, 5, 6, 7, 8, 9, 10, 11, 12, 13, 14, 15); min_samples_split = [2, 5, 10]; min_samples_leaf = [1, 2, 4]
ANN	Hidden_layer_sizes = [(50,), (100,), (50,50), (100,100)]; alpha= [0.0001,0.001,0.01]; size= c (1,2,3,4,5,6,7,8,9,10); model__learning_rate = [0.001, 0.01, 0.05]; epochs = [20, 30]; early_stopping = (monitor='loss', patience=5, restore_best_weights=True)
LR	c = (0.001, 0.01, 0.1, 1, 10, 100)
SVM	C = c (0.1, 1, 10, 100); sigma = c (0.001, 0.01, 0.1, 1)
XGB	n_estimators= [100, 200, 500]; max_depth= [3, 6, 9]; learning_rate= [0.01, 0.05, 0.1]; subsample= [0.8, 0.9, 1.0]; colsample_bytree= [0.8, 0.9, 1.0]
LGBM	objective = “binary”; metric = “binary_logloss”; boosting = “gbdt”; num_iterations = 100; learning_rate = 0.1; max_depth = 10; min_child_weight = 1; min_data_in_leaf = 20; feature_fraction = 0.3; bagging_fraction = 1; bagging_freq = 1; verbosity = −1

### 3.9 Shapley Additive Explanations (SHAP)

The SHAP analysis was utilized to enhance the interpretability of the ensemble machine learning models [48]. It is a model‐agnostic method on cooperative game theory that quantifies the contribution of each feature to the models predictions [49]. In this study, SHAP was employed to identify the most significant predictors of myopia and to evaluate their impact on the predicted outcomes. The analysis provided valuable insights into the role of individual risk factors, enabling a transparent understanding of the model's decision‐making process and supporting data‐driven interventions.

### 3.10 Evaluation Standards for Performance

Accuracy, precision, recall, F1‐score, geometric mean (g‐mean), and AUC are used to compare the performance of all applied prediction models to assess the effectiveness of ML‐based prediction models for myopia occurrences in individuals.

$$\begin{array}{c} \mathrm{Accuracy}=\frac{\mathrm{TP}+\mathrm{TN}}{\mathrm{TP}+\mathrm{FP}+\mathrm{TN}+\mathrm{FN}}\\ \mathrm{Sensitivity}=\frac{\mathrm{TP}}{\mathrm{TP}+\mathrm{FN}}\;\\ \mathrm{Specificity}=\frac{\mathrm{TN}}{\mathrm{TN}+\mathrm{FP}}\;\\ \text{f}1\;\text{score}\;=\;\frac{2*\mathrm{Sensitivity}*\mathrm{Specificity}}{\;\mathrm{Sensitivity}+\mathrm{Specificity}}\; \end{array}$$

The calculation formula of AUC is as follows:

$$\mathrm{AUC}=\int_{x=0}^{1}\text{TPR}\left(\mathrm{FP}R^{-1}\left(x\right)\right)\mathrm{dx}$$

$$\text{ROC}=\frac{\text{Sensitivity}+\text{Specificity}}{2}$$

**Figure 1:**
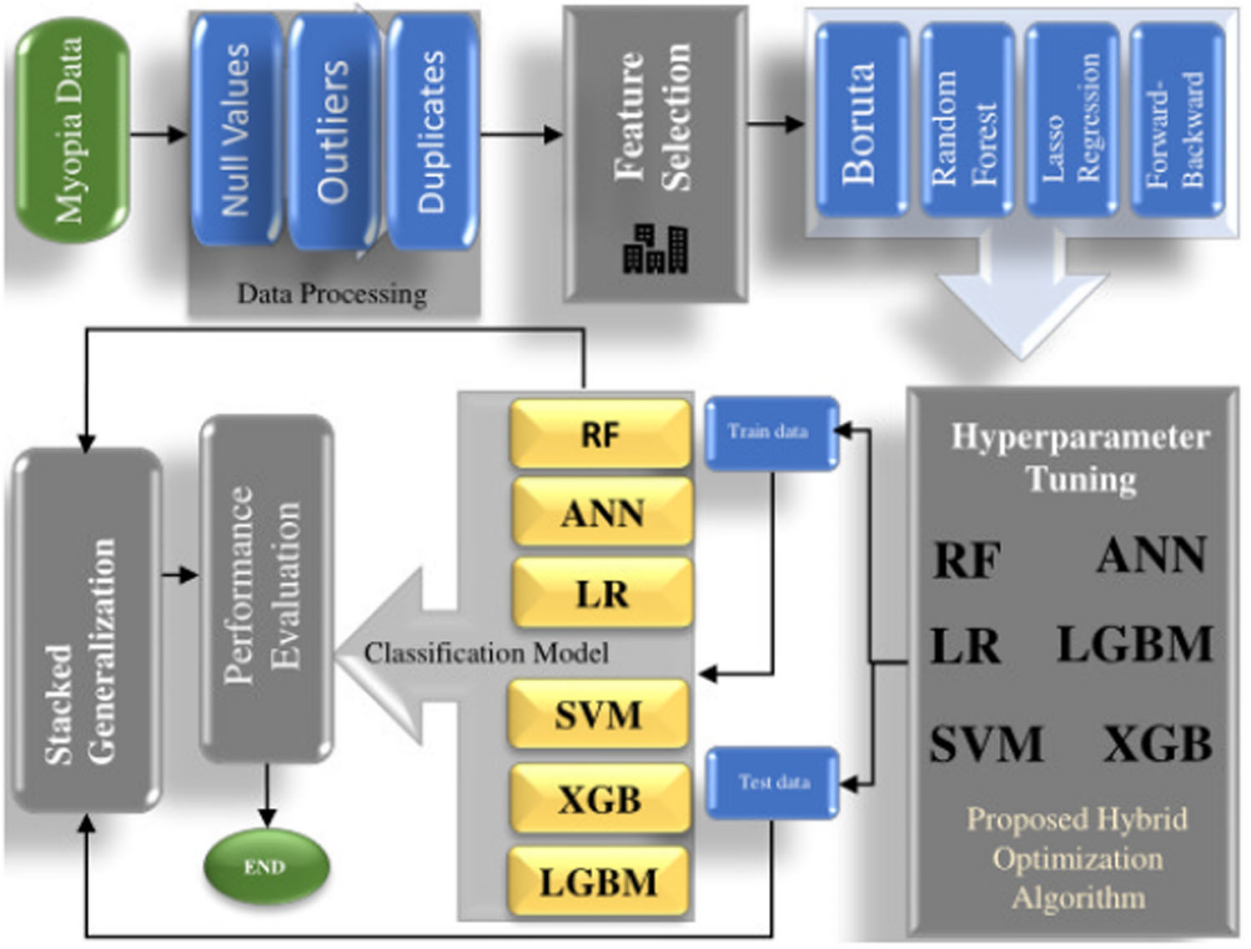
This is the overall workflow of the study.

The complete workflow of our study is shown in Figure 1.

### 3.11 Ethics Approval and Consent to Participate

This study is approved by the Institutional Animal, Medical Ethics, Biosafety, and Biosecurity Committee (IAMEBBC), University of Rajshahi, Rajshai‐6205, Bangladesh with approval number: 215/320/(69) | AMEBBC/|BSc. Written informed consent was obtained from all participants before their involvement in the study. Each participant was provided with a comprehensive explanation of the research objectives, procedures, potential risks, and benefits to ensure they fully understood the nature of their participation.

## 4 Results

### 4.1 Baseline Characteristics of the Participants

The study population consists predominantly of participants aged 21–23 years (55.1%), with those aged 24 years or older comprising 40.7% and those 20 years or younger making up only 4.3%. Females represent a larger portion (62.6%) compared to males (37.4%). A slight majority of participants live in urban areas (55.6%) versus rural areas (44.4%). Regarding their current residence, 43.6% live in halls, 31.5% in messes, 22.4% at home, and 2.3% in other types of accommodation. The majority of participants come from middle‐income families (87.7%), with 9.1% from poor families and 3.1% from rich families. Over half of the participants (55.1%) have a family member who also suffers from myopia, while 44.9% do not. Additionally, a small proportion of participants (7.4%) were born prematurely, whereas the vast majority (92.6%) was not. This demographic overview highlights key factors and potential influences related to the study's focus on myopia (see Table 2).

**Table 2:** Demographic information of the study participant's (*n* = 514).

Variable		Frequency (%)	Variable		Frequency (%)
Age	<=20 years	22 (4.3)	Current residual status	Hall	224 (43.6)
	21–23 years	283 (55.1)		Mess	162 (31.5)
	>=24 years	209 (40.7)		Home	115 (22.4)
Gender	Male	192 (37.4)		Others	12 (2.3)
	Female	322 (62.6)	Family income	Poor	47 (9.1)
Living place	Rural	228 (44.4)		Middle	451 (87.7)
	Urban	286 (55.6)		Rich	16 (3.1)
Born prematurely	Yes	38 (7.4)	Family history of myopia	Yes	283 (55.1)
	No	476 (92.6)		No	231 (44.9)

### 4.2 Identifying Risk Factors for Myopia Using Various Methods

To increase model accuracy, feature selection involves identifying and removing unnecessary, irrelevant, or inappropriate features from a data set. In this study, we employed four feature selection algorithms such as BFS method, LASSO regression, FBS, and RF to identify important features (see Table 3). The BFS algorithm identified 9 features, FBS revealed 7, RF identified 11, and LASSO regression revealed 7. Each method highlighted unique and significant features associated with myopia. Ultimately, we selected 12 common and unique features from these methods for further analysis.

**Table 3:** Feature selection results for identifying risk factors associated with myopia with their names, descriptions, and categorizations.

S/N	Name	Description	Categorization
1.	FmlyhisMy	Family history of myopia	Yes, no
2.	Eyepain	Suffering from eyepain	Yes, no, maybe
3.	SptiElede	Spending too much time on electronic devices?	Yes (> 6 h per day), no
4.	Glaucoma	Having glaucoma	Yes, no
5.	VisImp	Visual impairement	No, mild, moderate, severe, blind
6.	Injury	Eye injury	Yes, no, maybe
7.	Eyedsdr	Eye disorder	Yes, no
8.	Lightex	Screen light exposure	Yes, no, maybe
9.	NeWorAc	Near‐work activities	< 1 h, 1–3 h, 3–6 h and > 6 h
10.	Slepngtm	Sleeping time	4–6 h, 6–8 h, above 8 h
11.	SpOutAc	Sports and outdoor activities	< 0.5 h, 0.5–1 hiur, 1–2 h, > 2 h
12.	HihInDie	Higher Intake or dietary factors	Poor, middle, rich

After selecting the features, we checked for missing values and found none. Multicollinearity was assessed using VIF and correlation plot (see Figure S1), and the results indicated no multicollinearity among the 12 identified features. Further correlation analysis with the target variable revealed that the age of wearing contact lenses is highly correlated with myopia (see Figure S2). Conversely, gender was negatively correlated with myopia, indicating it has no significant effect, and was therefore excluded from our analysis. Finally, we selected 11 features for further study.

### 4.3 Analysis of ML‐Based Models' Respective Performances

The table presents the performance metrics of various models used to predict myopia, including LR, ANN, RF, SVM, XGB, LGBM, and the proposed stacking model (see Table 4). The confusion matrices are provided in Table S1, while the training and testing accuracy plots are shown in Figure S3. Among these models, the proposed stacking model demonstrated the highest accuracy (95.42%), recall (93.47%), precision (98.85%), F1‐score (96.08%), and AUC (0.979). This indicates that the stacking model outperforms the individual models, providing a more reliable and robust prediction of myopia. The high recall and precision values suggest that the stacking model is particularly effective in correctly identifying myopia cases while minimizing false positives. Overall, the results underscore the efficacy of ensemble learning techniques in improving predictive accuracy and reliability in myopia detection.

**Table 4:** Performance matrices of different models for predicting myopia.

Models	Accuracy	Recall	Precision	F1‐Score	AUC
LR	90.85	84.85	95.40	89.81	0.933
ANN	85.62	81.82	88.51	85.03	0.944
RF	88.31	83.05	91.58	87.81	0.959
SVM	90.91	86.44	93.68	89.91	0.937
XGB	87.01	86.90	89.02	87.95	0.959
LGBM	86.36	88.46	85.18	86.79	0.946
**Proposed stacking**	**95.42**	**93.47**	**98.85**	**96.08**	**0.979**

### 4.4 ROC and Precision Versus Recall Curve

**Figure 2:**
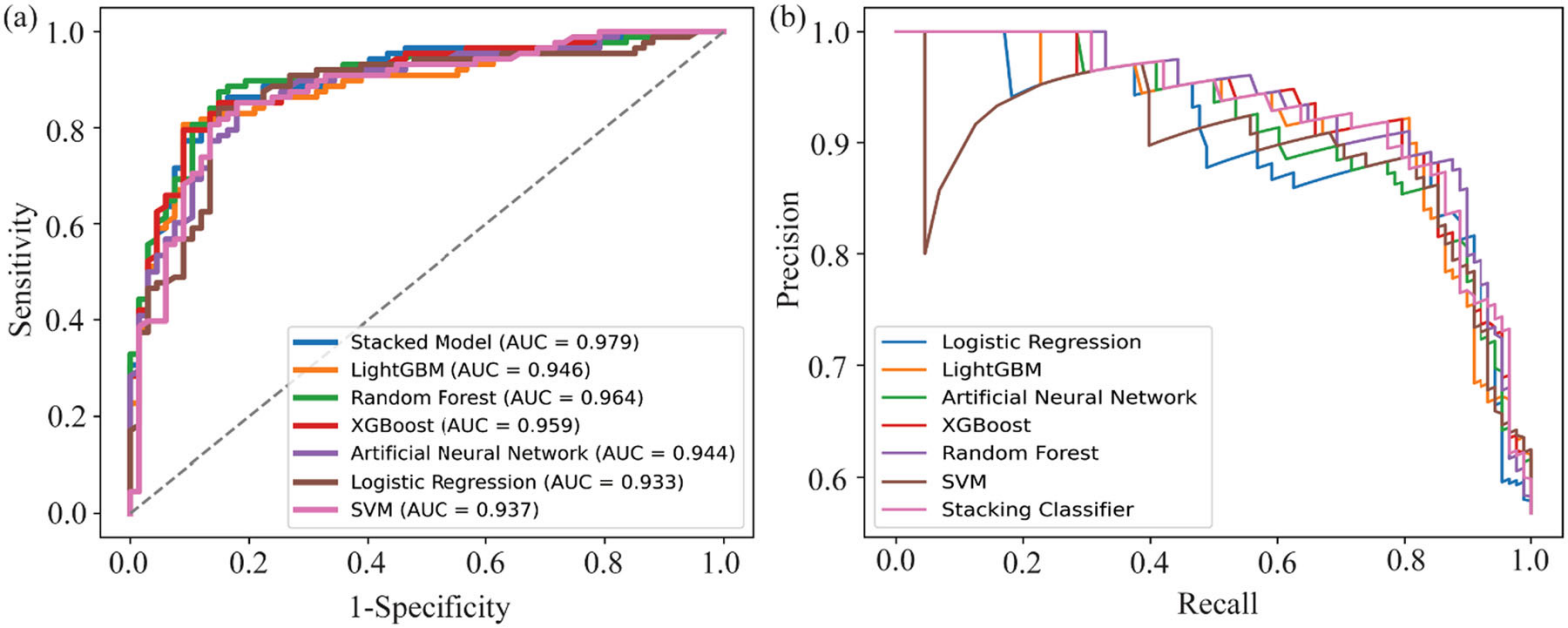
Line chart. (a) Discriminative ability of the six models compared using ROC and AUC, and (b) Precision versus Recall curves for the six predictive models.

The Receiver Operating Characteristic (ROC) curve is an effective method for assessing the performance of binary classification models. Figure 2a displays the ROC curves for five predictive models and the proposed stacking model. The AUC values are as follows: LR achieved 0.933, ANN 0.944, RF 0.964, SVM 0.937, XGB 0.959, LGBM 0.946, and the proposed stacking model 0.979. The proposed stacking model achieved the highest AUC, underscoring its superior ability to accurately differentiate between myopia and non‐myopia cases. Additionally, Figure 2b shows the precision versus recall curve, further evaluating model performance. Both curves demonstrated that our suggested stacking model outperforms the LR, ANN, RF, SVM, XGB, and LGBM.

### 4.5 Comprehensible Risk Factors for Myopia

**Figure 3:**
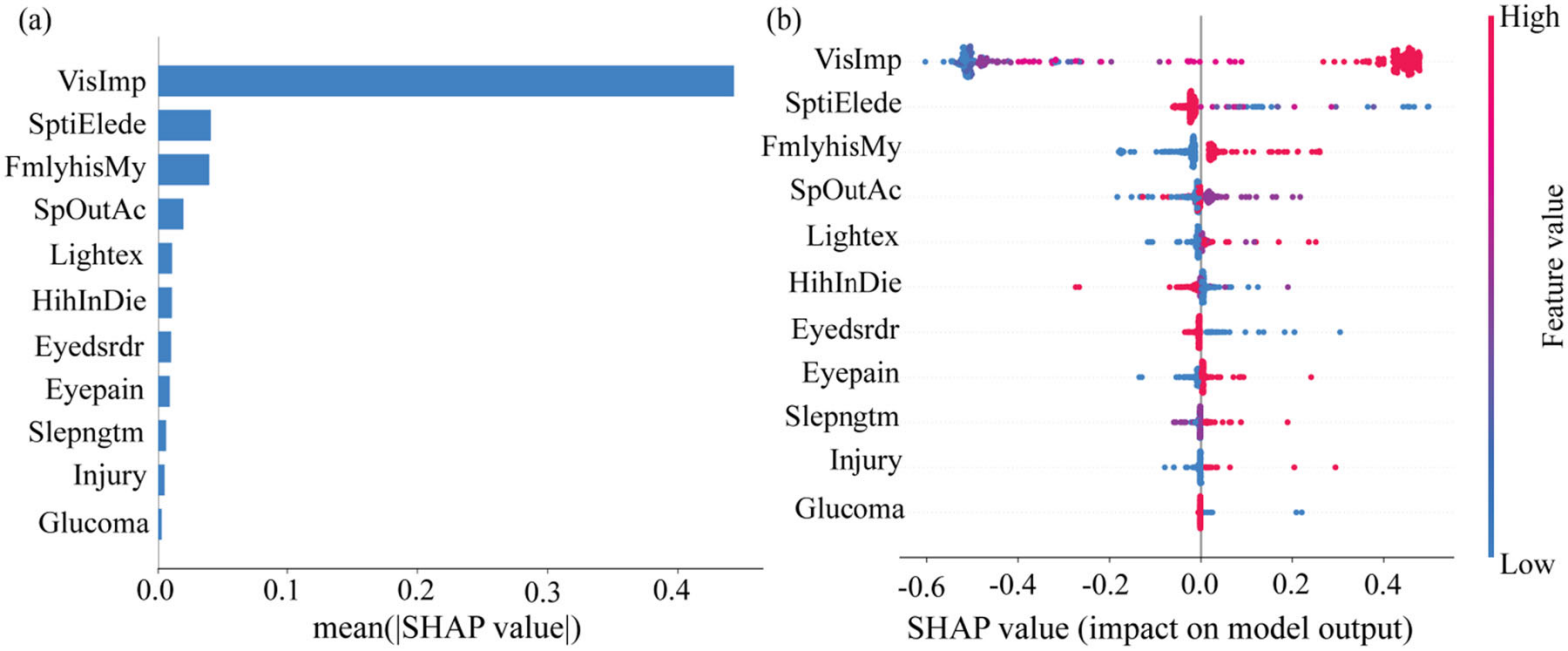
SHAP value. (a) Mean absolute SHAP values, to explain global risk factor importance, and (b) Local explanation summary, to reveal the direction of the relationship between a risk factor and outcome.

The SHAP (SHapley Additive exPlanations) summary plots displayed the impact of various features on the output of a machine learning model (see Figure 3). The mean SHAP values (see Figure 3a) indicate that Visual Impairment (VisImp) is the most influential feature, followed by Spending Too Much Time on Electronic Devices (SptiElede) and Family History of Myopia (FmlyhisMy). Features such as Sports and Outdoor Activities (SpOutAc) and Screen Light Exposure (Lightex) also show moderate importance, while Eye Injury (Injury) and Glaucoma have minimal impact. The SHAP value distribution (see Figure 3b) further reveals that high values of VisImp (red) consistently increase the model's predictions, while low values (blue) reduce them. Similar patterns are observed for SptiElede and FmlyhisMy, albeit with lesser impact. Features like SpOutAc and Lightex exhibit variable effects depending on their values. These findings underscore the significance of visual impairment and lifestyle factors in the predictive model. nt role of the age of first glasses use in myopia risk, along with other contributing factors.

## 5 Discussion

This study aimed to predict myopia among undergraduate students using ensemble machine learning techniques, with a focus on identifying key risk factors that contribute to myopia development. Our results demonstrated that ensemble models, particularly the stacking ensemble, achieved superior performance in terms of accuracy (95.42%), recall (93.42%), precision (98.85%), F1‐score (96.08%), and area under the curve (AUC) (0.979). These findings underscore the effectiveness of ensemble methods in improving predictive accuracy, which is crucial for early identification of myopia risk.

In comparison to previous studies [50, 51, 52, 53], our proposed model outperforms other machine learning approaches for myopia prediction. For example, studies using RF, XGBoost, and SVM achieved AUCs ranging from 0.84 to 0.98, while our stacking model achieved an AUC of 0.97, with the added advantage of SHAP analysis for feature interpretation.

The SHAP analysis revealed several key risk factors, including visual impairment, family history of myopia, excessive screen time, and insufficient outdoor activities. These factors have been consistently identified in the literature as important contributors to myopia development [14, 16, 54]. For instance, the association between family history of myopia and the likelihood of myopia has been well‐documented, as genetic factors play a significant role in refractive error development [55]. Similarly, visual impairment and its relationship with myopia are widely recognized, as individuals with uncorrected vision may have a higher risk of myopia progression [56]. Our study supports these findings and highlights their relevance in predicting myopia among undergraduate students.

The impact of excessive screen time on myopia risk has gained significant attention in recent years, with numerous studies linking prolonged near work activities, such as screen use, to myopia onset and progression [16, 57]. In our study, Spending Too Much Time on Electronic Devices was found to be one of the most influential features, which is consistent with current trends in lifestyle factors contributing to myopia [58]. The growing use of smartphones, computers, and other digital devices among young adults has been proposed as a contributing factor to the rise in myopia prevalence, especially in urban environments. Another important factor identified was insufficient outdoor activities, which has been shown to have a protective effect against myopia. Previous research suggests that outdoor activities, particularly exposure to natural light, may help delay the onset of myopia and slow its progression [59]. The variable effects of Screen Light Exposure (Lightex) in our study suggest that lifestyle modifications, such as reducing screen time and increasing outdoor exposure, could be effective strategies in mitigating myopia risk.

A key strength of this study is its reliance on a questionnaire‐based approach for data collection. Traditional myopia screening methods, such as clinical refraction tests and optical biometry, require specialized equipment and trained personnel, which can be resource‐intensive and inaccessible in many settings [60]. In contrast, a well‐designed questionnaire offers a cost‐effective, scalable, and easily deployable alternative for assessing myopia risk factors. Self‐reported lifestyle, behavioral, and genetic predisposition data provide valuable insights that complement clinical assessments and allow for large‐scale population‐based studies. Additionally, the use of machine learning techniques enhances the predictive capability of questionnaire‐based data, enabling early risk identification and targeted interventions even in resource‐limited settings [61]. By integrating behavioral, environmental, and genetic risk factors into predictive models, this study demonstrates that questionnaire‐based screening can serve as a viable alternative to traditional diagnostic methods, particularly in large academic populations where routine eye examinations may not be feasible.

Despite the promising results, several limitations should be acknowledged. First, the study was conducted in Dinajpur city, limiting the generalizability of the findings to other populations. The self‐reported nature of the questionnaire may have introduced biases, particularly in assessing daily activities and lifestyle factors. Additionally, the cross‐sectional design of the study does not allow for causal inferences, and further longitudinal studies are needed to explore how the identified risk factors influence myopia development over time. In terms of practical implications, the findings suggest that interventions aimed at reducing screen time, increasing outdoor activities, and addressing visual impairments may help prevent or mitigate myopia in undergraduate students. University health programs could incorporate these strategies to promote eye health, potentially through campaigns to raise awareness about myopia and its risk factors. Furthermore, the questionnaire‐based predictive model could be adopted as an initial screening tool to identify at‐risk individuals, prompting early clinical evaluations and personalized preventive measures.

## 6 Limitations of the Study

One limitation of this study is the relatively small sample size, as data were collected from three specific institutions in Dinajpur, which may not fully represent the broader undergraduate population of Bangladesh. Additionally, participation was voluntary, potentially leading to self‐selection bias and an uneven distribution of students across academic disciplines. This may have resulted in an overrepresentation of students who are more health‐conscious or particularly interested in vision‐related issues, thereby skewing the findings. Furthermore, due to the absence of comprehensive data on the total undergraduate population in Dinajpur, we could not fully assess the representativeness of our sample. Future studies should incorporate larger, more diverse samples and employ structured sampling methods to minimize bias and enhance the generalizability of the findings.

## 7 Conclusions

This study demonstrates the effectiveness of ensemble machine learning techniques, particularly the stacking ensemble model, in accurately predicting myopia among undergraduate students. By identifying key risk factors such as visual impairment, excessive screen time, and insufficient outdoor activities, the study underscores the importance of addressing these factors to reduce myopia risk. The stacking model's high performance (AUC = 0.97) highlights its potential for myopia prediction, with the added advantage of SHAP analysis for feature interpretation. While the findings offer valuable insights, further research, especially longitudinal studies, is needed to explore causal relationships and validate these results across diverse populations. The study also highlights the promising role of ensemble learning in medical diagnostics, and future work should focus on expanding predictive models to include additional environmental and genetic factors, with broader applications in healthcare beyond myopia prediction.

## Author Contributions

**Isteaq Kabir Sifat:** conceptualization, methodology, data curation, formal analysis, writing – original draft. **Tajin Ahmed Jisa:** conceptualization, data curation, methodology, formal analysis, writing – original draft. **Jyoti Shree Roy:** data curation, methodology, formal analysis. **Nourin Sultana:** data curation, formal analysis, visualization, software. **Farhana Hasan:** investigation, validation, methodology, visualization. **Md Parvez Mosharaf:** writing – review and editing, validation. **Md Kaderi Kibria:** conceptualization, methodology, formal analysis, supervision, validation, project administration, resources, writing – review and editing.

## Conflicts of Interest

The authors declare no conflicts of interest.

### 1 Transparency Statement

The lead author, Md. Kaderi Kibria, confirms that this manuscript provides an honest, accurate, and transparent account of the study being reported. No important aspects of the study have been omitted, and any discrepancies from the original study plan (or registered protocol, if applicable) have been clearly explained.

## Supporting information

Supplimentary file.

## Data Availability

The data that support the findings of this study are available from the corresponding author upon reasonable request. The datasets used and/or analyzed during the current study are available from the corresponding author upon reasonable request.
