# Supplementary material for: Prediction of Myopia Among Undergraduate Students Using Ensemble Machine Learning Techniques
Source: Health Sci Rep. 2025 May 26;8(5):e70874. doi: 10.1002/hsr2.70874 (PMC12106884; doi:10.1002/hsr2.70874)
Supplement: Supplementary file 1 — Supplimentary file. [file HSR2-8-e70874-s001.docx]

Prediction of Myopia among Undergraduate Students Using Ensemble Machine Learning Techniques

Supplementary file

| **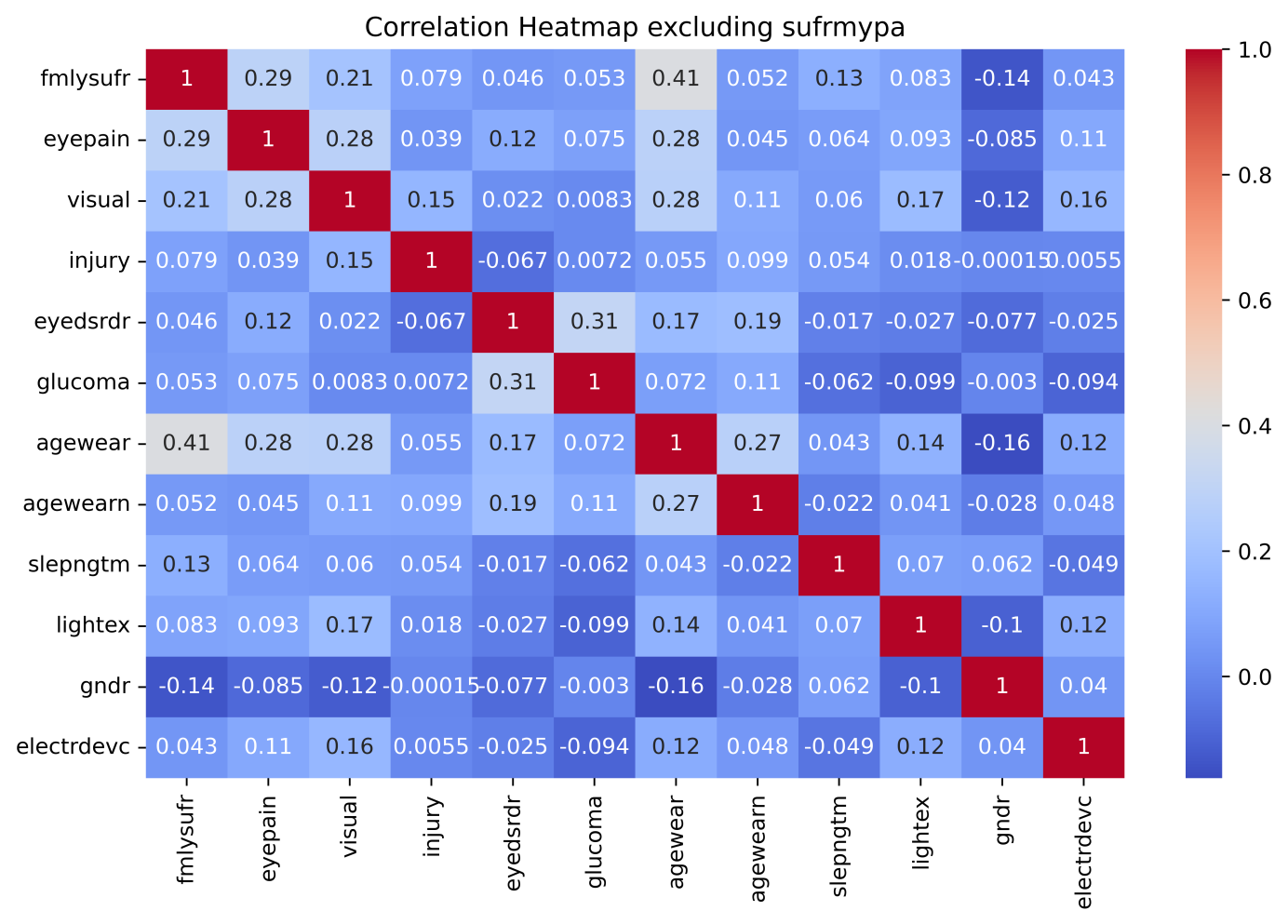**  **Fig S1:** Correlation plot among the identified 12 important features where color depth indicates the strength of the correlation. |
| --- |
| **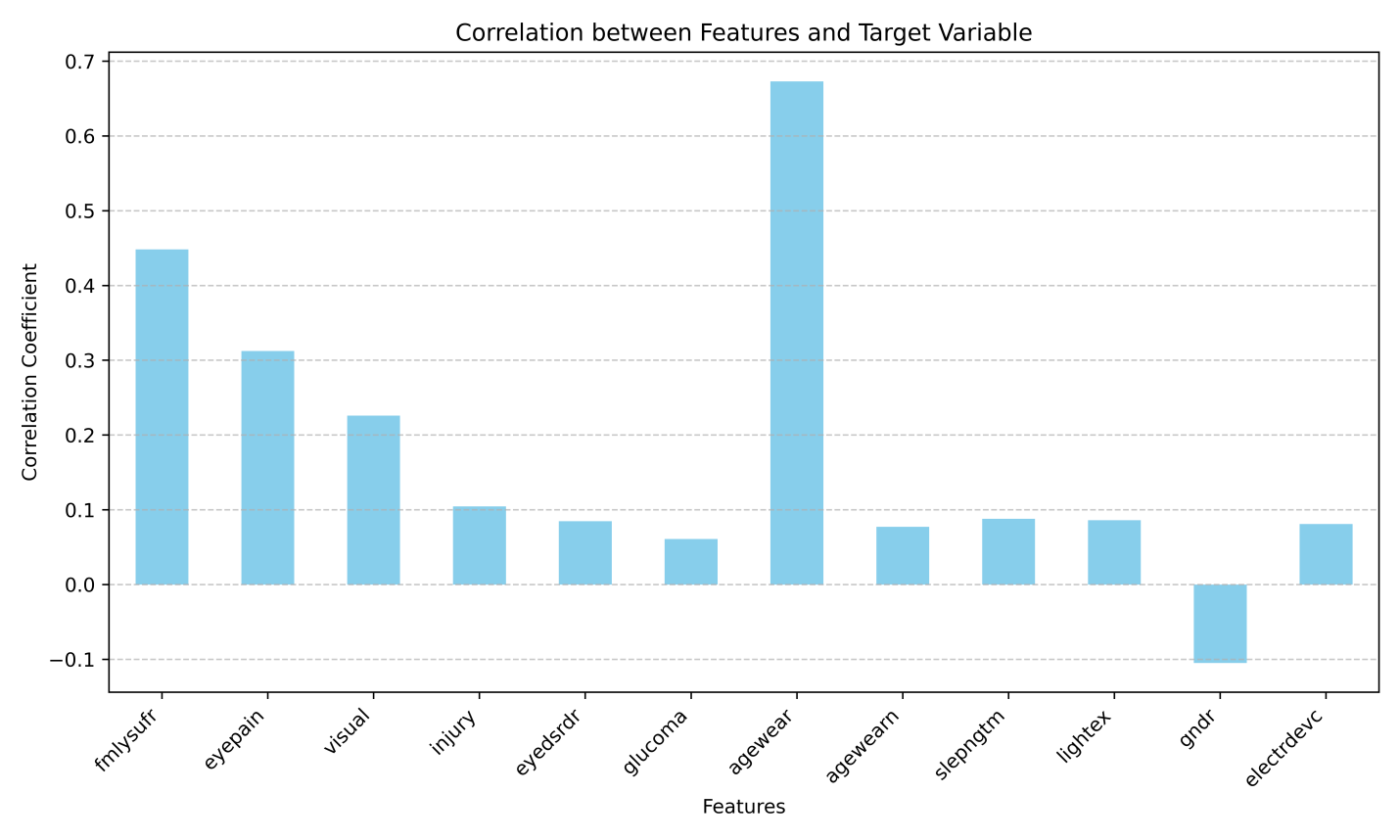Fig S2: Bar plot shown the correlation between identified features and target variable. The plot indicates that gender is negatively correlated with our target variable.** |

**Table S1: Confusion matrices of individual ML model and stacking ensemble model.**

|  |  | 0 | 1 |
| --- | --- | --- | --- |
| LR | 0 | 259 | 10 |
|  | 1 | 37 | 208 |
| ANN | 0 | 229 | 27 |
|  | 1 | 47 | 211 |
| RF | 0 | 270 | 43 |
|  | 1 | 87 | 427 |
| SVM | 0 | 229 | 32 |
|  | 1 | 70 | 444 |
| XGB | 0 | 20 | 43 |
|  | 1 | 67 | 434 |
| LGBM | 0 | 50 | 25 |
|  | 1 | 59 | 430 |
| Stacking Model | 0 | 90 | 16 |
|  | 1 | 33 | 471 |

| 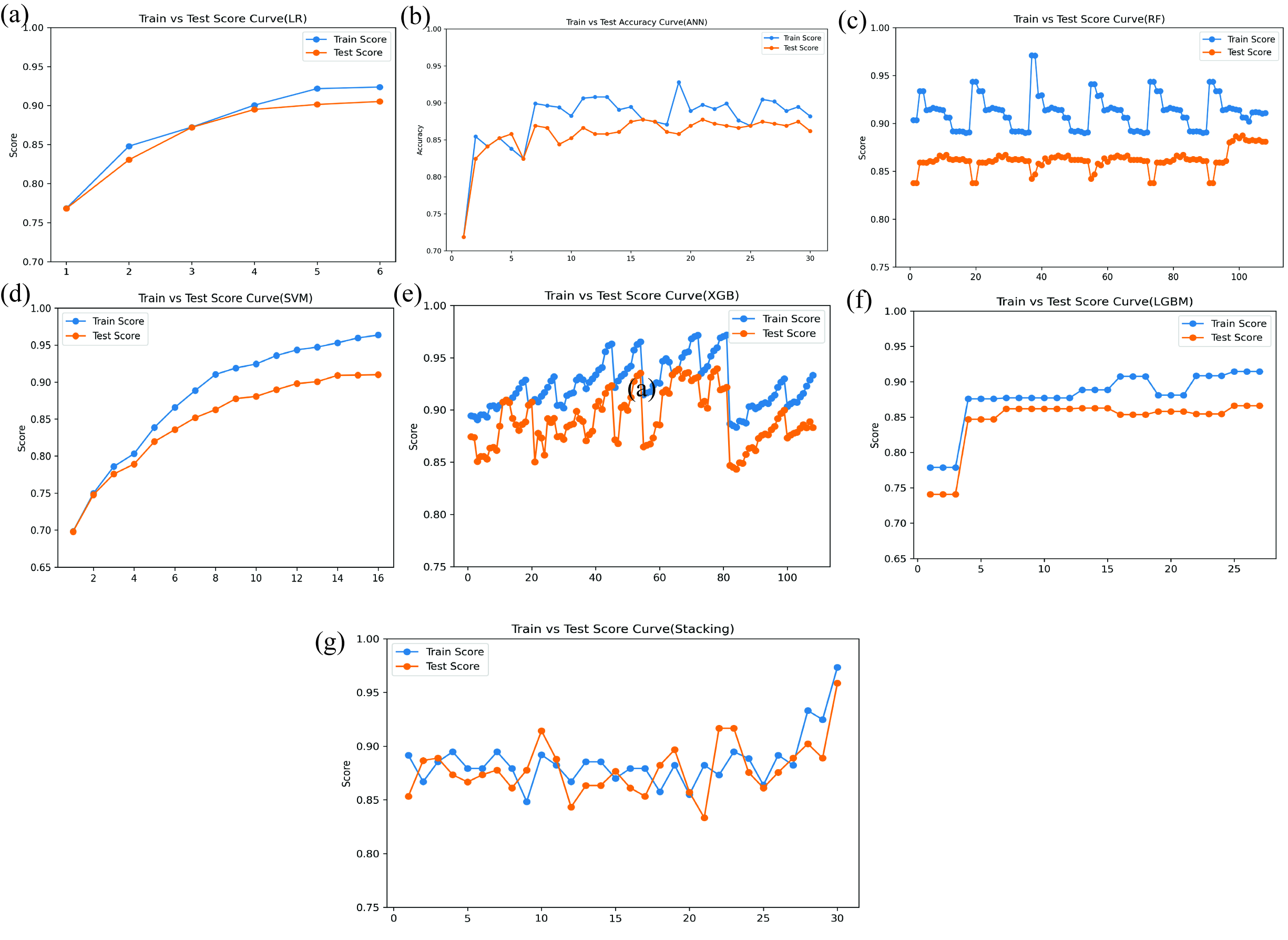  **Fig S3: Training and testing accuracy plot the individual ML methods (a-f) and stacking ensemble model (g).** |
| --- |
